# The Efficacy of Tranexamic Acid in Anterior Cruciate Ligament Reconstruction: A Systematic Review and Meta‐Analysis

**DOI:** 10.1155/aort/9180643

**Published:** 2026-04-28

**Authors:** Ben Nagy, Thomas Cho, Briggs Hehl, Jason Suh, Jiayong Liu

**Affiliations:** ^1^ Department of Orthopedic Surgery, University of Toledo Medical Center, Toledo, 43614, Ohio, USA, utoledo.edu

**Keywords:** anterior cruciate ligament, outcomes, reconstruction, tranexamic acid

## Abstract

**Introduction:**

Arthroscopic reconstruction of the anterior cruciate ligament (ACL) is a well‐established surgical intervention following ligament compromise. Administration of tranexamic acid (TXA), an antifibrinolytic agent, has been trialed to improve a variety of surgical outcomes following ACL reconstruction. The objective of this study is to further analyze the effect of TXA on postoperative drain output, functional scores, and overall knee functionality following ACL reconstruction.

**Methods:**

Literature retrieval was accomplished using PubMed and Google Scholar up until May 2025. Studies were included if ACL reconstruction outcomes were compared between TXA and control groups and if the relevant outcomes were reported. Review Manager Web was used for statistical analysis, and *p* values ≤ 0.05 were considered statistically significant.

**Results:**

14 studies were ultimately included in this meta‐analysis, totaling 1328 patients: 689 in the TXA group and 639 in the control group. Regarding VAS scores, significant differences were seen at 1 day (*p* = 0.02), 1 week (*p* < 0.001), 2 weeks (*p* = 0.02), and 4 weeks postoperation (*p* = 0.01) in favor of the TXA group. Regarding range of motion (ROM), a significant difference was found at 2 weeks postoperation (*p* = 0.005) in favor of TXA. Regarding the drain output measured 1 day postoperatively, the TXA group had a significantly lower drain output than the control group (*p* < 0.001). No significant differences were found in hemoglobin levels measured 1 day postoperation, Lysholm score at 1 and 3 months postoperation, VAS at 6 weeks postoperation, and ROM at 1 day and 4 weeks postoperation.

**Conclusion:**

The TXA group demonstrated significantly better drainage output, pain scores, and ROM in the early postoperative period. The use of TXA in ACL reconstruction appears to yield promising results, leading to a more prompt and efficient rehabilitation process and an overall reduction in pain for patients.

## 1. Introduction

The anterior cruciate ligament (ACL) is the most commonly injured ligament of the knee, with reports establishing injuries in 1 in 3500 individuals per year in the United States [1]. Injuries are most prevalent in collegiate athletics, specifically women’s gymnastics and men’s football, with a great portion of injuries occurring in women [2].

Arthroscopic reconstruction of the ACL is a well‐established surgical intervention following ligament compromise. Despite the benefits of arthroscopy, postoperative joint fluid accumulation and pain remain barriers to recovery. Intraoperative drains have been implemented with the goal of reducing hemarthrosis and improving postoperative outcomes. However, several studies have analyzed their efficacy with many showing no added benefit [3–7]. More recently, perioperative or intraoperative administration of tranexamic acid (TXA), an antifibrinolytic agent, has been trialed as a method for improving a variety of surgical outcomes following ACL reconstruction (ACLR). TXA is a synthetic derivative of lysine and possesses antifibrinolytic effects by reversibly blocking lysine‐binding sites on plasminogen molecules, inhibiting its interaction with fibrin. TXA has been used as a prophylactic measure to prevent blood loss and limit allogeneic transfusions in patients undergoing surgery [8].

There have been several studies in the literature that have compared postoperative outcomes in patients undergoing ACLR between those who have received TXA and those who have not. However, there have been newer studies published regarding this topic, and these data are yet to be included in an updated meta‐analysis. Therefore, the objective of this study is to provide a comprehensive and updated systematic review and meta‐analysis that further analyzes the effect of TXA on postoperative drain output, functional scores, and overall knee functionality following ACLR.

## 2. Methods

The Preferred Reporting Items for Systematic Reviews and Meta‐Analyses (PRISMA) guideline was used for this study [9].

### 2.1. Literature Search

Literature retrieval was accomplished using PubMed and Google Scholar. Data retrieval was carried out until May 2025, and key words for literature search included “tranexamic acid,” “anterior cruciate ligament,” “reconstruction,” and “outcomes.”

### 2.2. Inclusion and Exclusion Criteria

Study criteria included published randomized controlled trials (RCT) as well as retrospective and prospective comparison studies. Studies were included if ACLR outcomes were compared between TXA administration and control groups. All systemic administration methods of TXA were included, such as intravenous and intra‐articular methods. Studies were included if data contained outcome measures related to postoperative visual analog scale (VAS) scores, Lysholm scores, hemoglobin levels, drain output, and range of motion (ROM) at various timelines. A study was excluded if TXA was compared to other perioperative agents that went beyond control measures and/or if the study was a review article, case report, or biomechanical study. One study included both intravenous and intra‐articular administration methods for the TXA samples [10]. Since this meta‐analysis was to include both administration types among studies, the intravenous and intra‐articular groups were combined into a single group to use for statistical analyses according to methods outlined in the Cochrane handbook [11].

### 2.3. Assessment of Study Quality

The inclusion and exclusion criteria mentioned above were applied to retrieved articles by each author independently. For the assessment of study quality in RCTs, the Cochrane Risk of Bias Tool was used, which can be done with the Review Manager Web software. Each study was scored as a low risk, unclear risk, or high risk of bias based on the following parameters: random sequence generation (selection bias), allocation concealment (selection bias), blinding of participants and personnel (performance bias), blinding of outcome assessment (detection bias), incomplete outcome data (attrition bias), selective reporting (reporting bias), and other bias [12]. For all other nonrandomized studies, the Newcastle–Ottawa scale was used [13].

### 2.4. Data Collection

Data that were collected from each included study are as follows: first author, publication year, journal, sample sizes, drain output at 1 day, Lysholm score at 1 month, Lysholm score at 3 months, VAS score at 1 day, VAS score at 1 week, VAS score at 2 weeks, VAS score at 4 weeks, VAS score at 6 weeks, ROM at 1 day, ROM at 2 weeks, ROM at 4 weeks, and hemoglobin at 1 day. For the outcomes mentioned above, those were referring to postoperative results, and the data that were collected were stored in a shared Excel sheet.

### 2.5. Statistical Analysis

Statistical tests were carried out using Review Manager Web. All postoperative outcomes of interest were continuous variables. Therefore, they were presented as mean ± standard deviation (SD). An inverse variance method with a mean difference (MD) was used. Regarding the assessment of heterogeneity, the I^2^ statistic was used for each analysis with the following interpretation: 0%–40% (might not be necessary), 30%–60% (may represent moderate heterogeneity), 50%–90% (may represent substantial heterogeneity), and 75%–100% (considerable heterogeneity) [14]. If *I* ^2^ ≤ 50%, a fixed effect analysis model was used. If *I* ^2^ > 50%, a random effects analysis model utilizing the DerSimonian and Laird and Wald‐type confidence interval (CI) methods was used. A *p* value ≤ 0.05 was considered statistically significant, and all results were presented as a forest plot with a 95% CI.

## 3. Results

### 3.1. Overview of Included Studies

**FIGURE 1:**
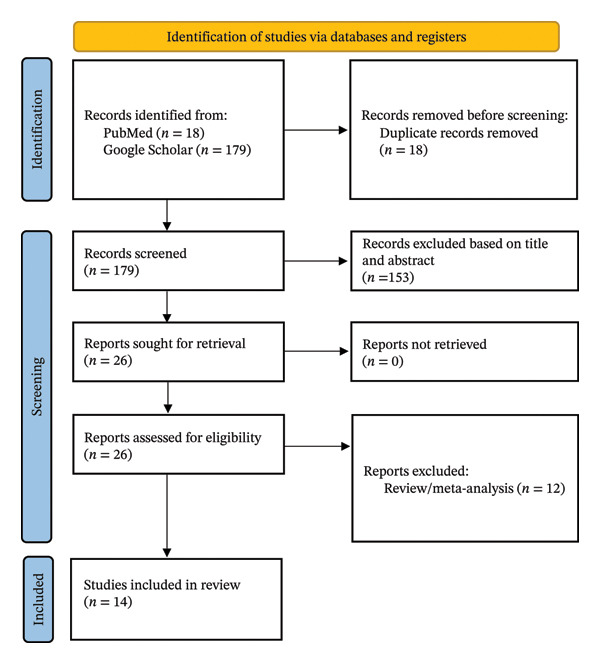
PRISMA 2020 flow diagram of the study selection process.

Fourteen studies were ultimately included in this study [10, 15–27] (Figure 1). 12 studies were RCTs, one was a retrospective cohort study, and one was a prospective cohort study. There was a total of 1328 patients included in this meta‐analysis, with 689 patients in the TXA group and 639 in the control group (Table 1).

**TABLE 1:** Baseline characteristics of the included studies.

Author (year)	Journal	Study design	TXA	Control	Outcomes
Cen et al. (2024) [15]	J Orthop	RCS	40	40	Lysholm 3‐month, VAS 1‐day, VAS 2‐week, VAS 6‐week, ROM 2‐week
Chiang et al. (2019) [16]	Arthroscopy	RCT	151	149	Drain output 1‐day, VAS 4‐week, ROM 4‐week
Felli et al. (2019) [17]	Arthroscopy	RCT	40	40	Drain output 1‐day, Lysholm 1‐month, Lysholm 3‐month, ROM 1‐day, ROM 2‐week, ROM 4‐week, hemoglobin 1‐day
Fried et al. (2021) [18]	Arthroscopy	RCT	54	53	VAS 1‐week, VAS 6‐week
Kalina et al. (2021) [19]	Acta Chir Orthop Traumatol Cech	RCT	25	18	Lysholm 1‐month, Lysholm 3‐month, VAS 1‐day, VAS 4‐week, hemoglobin 1‐day
Karaaslan (2015) [20]	Am J Sports Med	RCT	53	52	ROM 1‐day
Kittijarukajon et al. (2024) [21]	Chonburi Hospital Journal	RCT	30	30	Drain output 1‐day, VAS 1‐day, VAS 1‐week, VAS 2‐week, ROM 2‐week, ROM 4‐week, hemoglobin 1‐day
Lee et al. (2020) [22]	Orthop J Sports Med	RCT	23	24	VAS 1‐day, hemoglobin 1‐day
Ma et al. (2021) [10]	BMC Musculoskelet Disord	RCT	80	40	Drain output 1‐day, Lysholm 1‐month, VAS 1‐week, VAS 2‐week, VAS 4‐week
Mikic et al. (2024) [23]	Cir Cir	RCT	62	62	VAS 1‐day, VAS 1‐week, VAS 6‐week
Mousavi et al. (2023) [24]	Eur J Orthop Surg Traumatol	RCT	30	31	VAS 1‐day, VAS 1‐week, VAS 2‐week, VAS 4‐week
Pande and Bhaskarwar (2019) [25]	Int J Res Orthop	RCT	24	24	Lysholm 3‐month, ROM 2‐week
Shawky et al. (2024) [26]	Benha Medical Journal	RCT	25	25	Drain output 1‐day, hemoglobin 1‐day
Yuncu et al. (2025) [27]	Arch Trauma Res	PCS	52	51	Drain output 1‐day, VAS 1‐day, hemoglobin 1‐day

### 3.2. Functional Outcomes

Regarding Lysholm score, there were no significant differences observed at both 1 month (MD: 1.99; 95% CI: −0.94–4.93; *p* = 0.18; Figure 2(a)) and 3 months postoperation (MD: 2.89; 95% CI: −2.33–8.11; *p* = 0.28; Figure 2(b)).

FIGURE 2Forest plot of the Lysholm score at (a) 1 month and (b) 3 months postoperatively.

**Figure:**
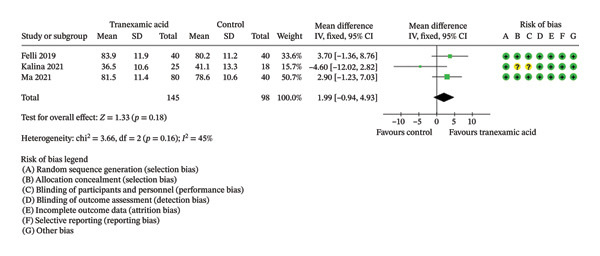
(a)

**Figure:**
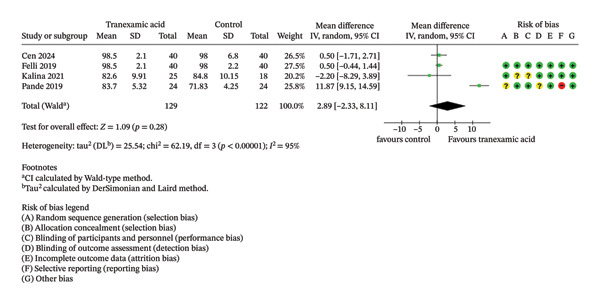
(b)

Regarding VAS scores, significant differences were seen at 1 day (MD: −0.92; 95% CI: −1.72 to −0.13; *p* = 0.02; Figure 3(a)), 1 week (MD: −0.57; 95% CI: −0.90 to −0.24; *p* < 0.001; Figure 3(b)), 2 weeks (MD: −0.63; 95% CI: −1.16 to −0.11; *p* = 0.02; Figure 3(c)), and 4 weeks postoperation (MD: −0.23; 95% CI: −0.41 to −0.06; *p* = 0.01; Figure 3(d)) in favor of the TXA group, while no significant difference was found at 6 weeks postoperation (MD: −0.33; 95% CI: −1.02 to 0.36; *p* = 0.35; Figure 3(e)).

FIGURE 3Forest plot of the visual analog scale (VAS) pain score at (a) 1 day, (b) 1 week, (c) 2 weeks, (d) 4 weeks, and (e) 6 weeks postoperatively.

**Figure:**
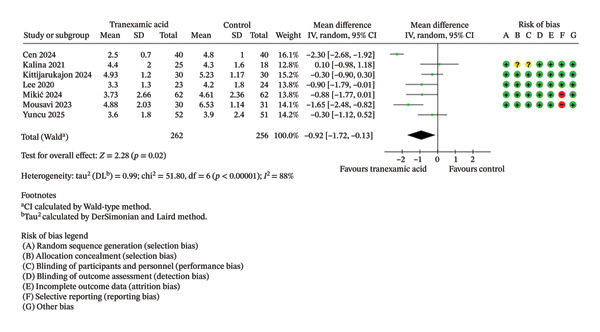
(a)

**Figure:**
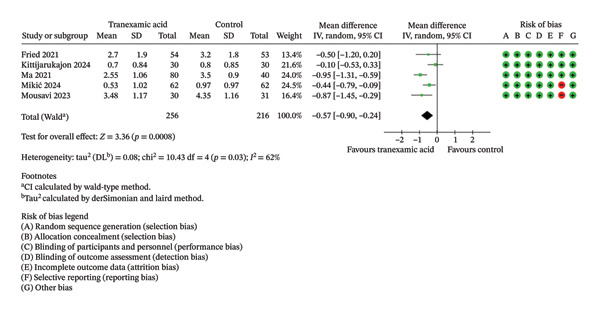
(b)

**Figure:**
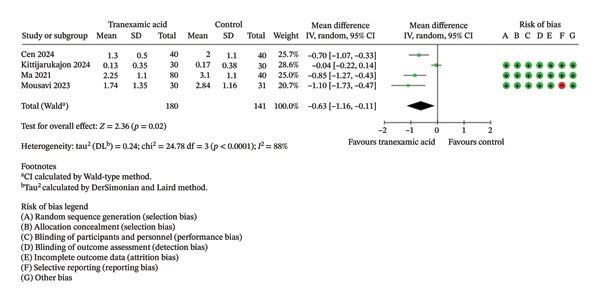
(c)

**Figure:**
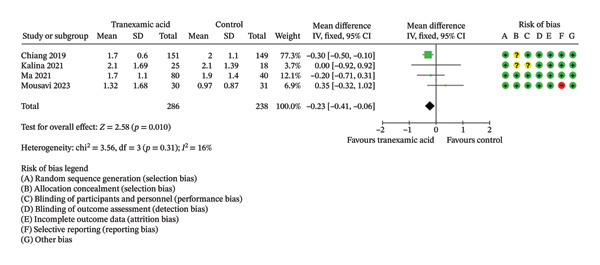
(d)

**Figure:**
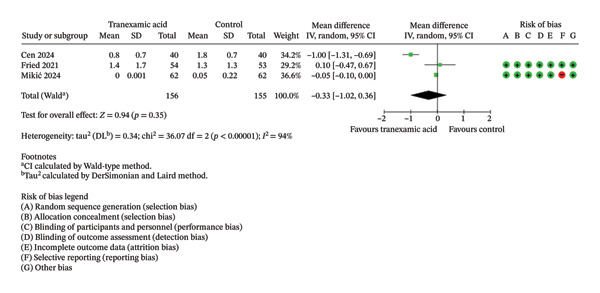
(e)

Regarding ROM, a significant difference was found at 2 weeks postoperation (MD: 5.86; 95% CI: 1.75–9.98; *p* = 0.005; Figure 4(a)) in favor of TXA, while no significant differences were found at 1 day (MD: 2.88; 95% CI: −0.15 to 5.91; *p* = 0.06; Figure 4(b)) and 4 weeks postoperation (MD: 4.06; 95% CI: −0.11–8.23; *p* = 0.06; Figure 4(c)).

FIGURE 4Forest plot of the range of motion (ROM) at (a) 1 day, (b) 2 weeks, and (c) 4 weeks postoperatively.

**Figure:**
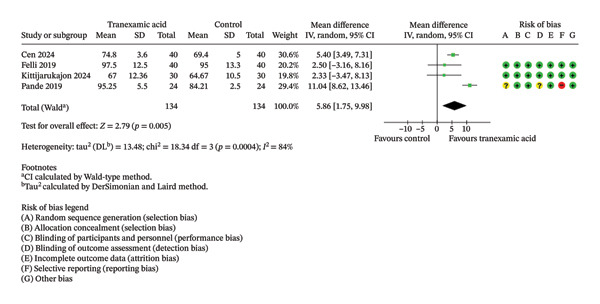
(a)

**Figure:**
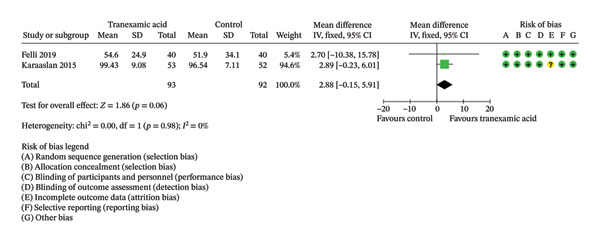
(b)

**Figure:**
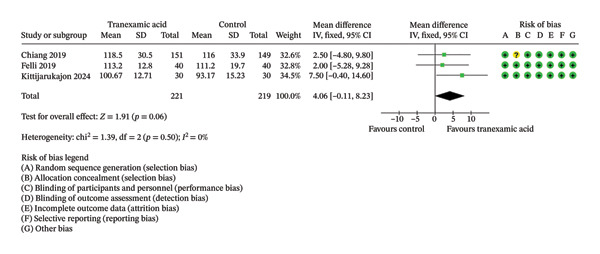
(c)

### 3.3. Other Outcomes

**FIGURE 5:**
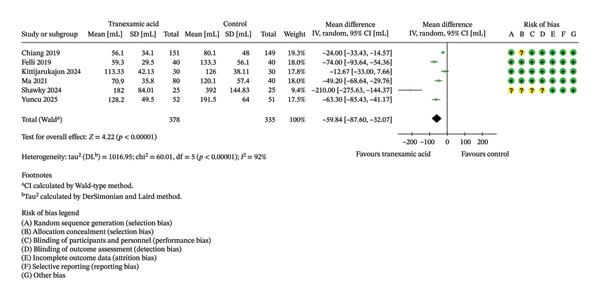
Forest plot of the drain output on postoperative day 1.

Regarding drain output measured 1 day postoperation, the TXA group had a significantly lower drain output compared to the control group (MD: −59.84; 95% CI: −87.60 to −32.07; *p* < 0.001; Figure 5).

**FIGURE 6:**
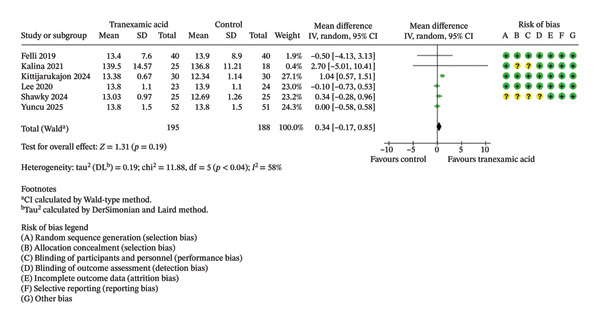
Forest plot of the hemoglobin level on postoperative day 1.

Regarding hemoglobin levels measured 1 day postoperation, no significant difference was found between the TXA and control groups (MD: 0.34; 95% CI: −0.17–0.85; *p* = 0.19; Figure 6).

## 4. Discussion

This review investigated the effect of TXA on postoperative outcomes and overall knee functionality following ACLR. Findings from this study support the use of TXA as it relates to decreased drain output, decreased VAS score, and increased ROM. These factors offer a variety of clinical significance. Decreasing drain output, as seen in the TXA group in this study, aids in potentially decreasing the risk of prolonged wound drainage and therefore prolonged hospitalization [28]. This proves crucial to limiting postoperative infection risks, as each day of postoperative wound drainage increases the risk of infections significantly by 29% [28]. Prolonged wound drainage postoperatively has been correlated to lower postoperative hemoglobin levels [29]. The result of this study is in accordance with other meta‐analysis studies that have shown a significant reduction in drain output in favor of TXA [30, 31]. The use of drains in knee surgery and ACLR is controversial. The goal of postoperative drain usage is to remove accumulated hemarthrosis and fluid in the joint space, reducing swelling in hopes of relieving knee pressure, pain, and stiffness. Studies have demonstrated its effectiveness showing decreased use of opioid medications 6 h postoperatively, suggesting a pain benefit for patients in the early recovery process [32]. Other evidence has suggested no postoperative recovery benefits and worse postoperative pain scores associated with drain usage [3–7]. This study showed that TXA results in a decreased drain output 24 h postoperatively, potentially serving as an alternative to drains. Decreased drain output may be the result of TXA’s reduction in the formation of hemarthrosis and fluid collection in the joint space, the same factors that drains are attempting to eliminate. This may be correlated to TXA’s effectiveness in reducing pain and stiffness postoperatively, evident by reduced VAS scores and higher ROM values. Additionally, TXA is noninvasive, eliminating the acquired infection risk associated with an inserted drain device.

As demonstrated in this study, TXA administration during ACLR established similar hemoglobin levels 24 h after surgery compared to the control group. Hemoglobin serves as the body’s primary transporter of oxygen. Oxygen plays an important role in wound healing by acting against pathogens and aiding in wound granulation, cell proliferation, and vascularization, thus aiding the wound healing process, along with fighting off infection [33]. Although this result was not significant, it is still an important factor to consider in future research and clinical practice.

The results of this study showed that TXA administration during ACLR had significantly lower VAS scores at 1 day, 1 week, 2 weeks, and 4 weeks postoperation, although no significant difference was seen at 6 weeks postoperation. Similar results were reported in other meta‐analysis studies [30, 31, 34]. Lower VAS scores with TXA can possibly be attributed to its anti‐inflammatory effects, demonstrated by lower C‐reactive protein and interleukin‐6 following orthopedic operations compared to control groups [35]. Lower VAS scores could be an indication to minimize opioid use for pain management, which can contribute to mitigating the risk of developed opioid dependence following ACLR. This is rather important, as current data suggest 1 in 500 opioid‐naive patients develop opioid dependence or overdose in a 5‐year follow‐up window following a postsurgery opioid prescription [36]. Regarding the nonsignificant difference seen at 6 weeks postoperation, the authors believe this is most likely due to the acute inflammatory response and hemarthrosis resolving by week 6 and there afterward, leading to similar pain levels between the two groups.

Additionally, compared to control groups, this study showed that those who received TXA demonstrated greater postoperative ROM at 2 weeks postoperation compared to the control group, although no significant differences were found at 1 day and 4 weeks. These results agree with other studies found in the literature [30, 31]. The results suggest that TXA improves ROM in the early postoperative period but not in the long term, and similar to VAS score, this is most likely due to TXA having the biggest impact on reducing acute hemarthrosis and inflammation, which are primarily seen in early phases of recovery. VAS scores and ROM may go hand‐in‐hand, as postoperative knee pain and knee ROM values may demonstrate reciprocal benefit, as lesser pain scorers can aid in increased ROM and greater ROM can lead to less postoperative pain. These improved pain and movement metrics can be vital in rehabilitation processes, especially for athletes. It is reasonable to assume that lower pain levels immediately postsurgery will allow an athlete to progress through rehabilitation sooner and more efficiently in order to return to their preoperation level of play. The literature showed that quadricep strength was also significantly higher in the TXA group 15 days postsurgery, which can further contribute to excelling in the knee rehabilitation process soon after surgery [15, 17].

Despite the benefits previously mentioned, it is important to highlight the concerns raised regarding the potential for dose‐dependent cytotoxic effects of TXA. Laboratory studies have shown that TXA can exhibit toxic effects on chondrocytes, tenocytes, and synoviocytes with concentrations above 20 mg/mL, with further studies showing atypical morphology, reduced cellular adhesion, and increased chondrocyte death in human articular cartilage at this concentration range [37, 38]. The proposed mechanism of chondrotoxicity seems to involve apoptosis mediated by endoplasm reticulum stress activation, generation of reactive oxygen species, and mitochondrial membrane depolarization [39]. Current evidence in the basic science literature suggests that TXA administration at concentrations of 20 mg/mL or less is expected to be safe, although it is important to note that there are no published clinical trials at this time that report toxic effects from TXA on articular tissues in human patients [37, 38].

This study found no significant differences in Lysholm score at both 1 and 3 months postoperation. One meta‐analysis found a significant difference in favor of TXA at 4–6 weeks, and another study found a significant difference in favor of TXA at 2 weeks and 4–6 weeks but not at 12 weeks [30, 34]. The authors believe that the differences seen at around 1 month postoperation is most likely due to the addition of newer studies in this meta‐analysis that might offer a new perspective on the impact of TXA on postoperative functionality. However, the results from this study seem to agree with the current literature that there is no significant difference in patient‐reported outcomes 6 weeks after the operation.

In the postoperative period, especially with the use of an antifibrinolytic agent, it is important to discuss thromboprophylaxis. In the included studies, there was a wide range of prophylactic methods, and these were not analyzed as there was no standardization in protocols across included studies. Of note, none of the included studies mentioned any instance of deep vein thrombosis, pulmonary embolism, or any other instance of blood clots, potentially suggesting that each of these prophylactic methods successfully prevented thrombosis. Across studies, there was no evidence suggesting a thrombotic consequence of TXA utilization.

It is important to note the limitations of the current study. The administration route of TXA between intravenous or intra‐articular was not separated, but current data suggest no differences in outcomes between the two techniques [10, 40–42]. Additionally, time to surgery was not compared between TXA and control groups, which could play a role in postoperative measurements. Postoperative differences in pain evaluation and ROM could be dependent on the use of specific grafts and techniques, and the influence of these factors was not isolated or analyzed. The majority of the studies utilized a hamstring autograft, being a semitendinosus or bicep femoris tendon, or a gracilis autograft. One study utilized peroneus longus tendon autografts [27]. Another study utilized a bone‐patellar tendon‐bone autograft [18]. Based on literature review, it is important to note that bone‐patellar tendon‐bone autografts have demonstrated higher pain ratings in the acute postoperative period compared to autologous hamstring grafts, while peroneus longus autografts have established similar pain and function scores postoperatively when compared to autologous hamstring grafts [43, 44]. The authors would also like to note that the many of the cases seen in this cohort were not isolated ACL repairs, with some undergoing meniscal repairs and meniscectomies. Unfortunately, the majority of included studies did not subgroup their patients by isolated versus accessory procedures, and thus this study included both types of cases. Therefore, the inclusion of concomitant procedures could have possibly influenced the results. Postoperative pain control was also not analyzed, and stronger prescriptions (i.e., oxycodone vs. acetaminophen) may have an impact on postoperative pain and ROM scores. Lastly, instances of high risk of bias in selective reporting were found in three studies [23–25].

## 5. Conclusion

The TXA group established significantly better postoperative outcome measures, including drain output, VAS score, and ROM in the early postoperative period, although the effects seem to taper off in the long term. These measures may further contribute to decreased hospital stay and risks of infection, as well as wound healing. Lower postoperative pain scores can decrease the use of opioid prescriptions and, along with higher postoperative ROM values, can lead to a more prompt and efficient rehabilitation process overall.

## Funding

The authors received no specific funding for this work.

## Conflicts of Interest

The authors declare no conflicts of interest.

## Data Availability

The data that support the findings of this study are available on request from the corresponding author.
